# Neuropsychiatric disturbance detecting polycythemia vera myelofibrosis: a case report and literature review

**DOI:** 10.3389/fneur.2023.1253468

**Published:** 2023-09-22

**Authors:** Li Li, Min Zhou, Yun-Qin Wu, Wei-Nv Fan, Da Li

**Affiliations:** Department of Neurology, Ningbo No 2 Hospital, Ningbo, Zhejiang, China

**Keywords:** post-polycythemia vera myelofibrosis (post-PV MF), neuropsychiatric symptoms (NPS), chorea, microcirculation disorder, Janus kinase 2 (JAK2)

## Abstract

**Background:**

Neuropsychiatric disturbances and chorea are less recognized consequences of polycythemia vera (PV), and their role in post-PV myelofibrosis (MF) has not been reported. Clinical features that predict post-PV MF lack specificity.

**Case presentation:**

We describe an elderly patient with PV who developed acute-onset reversible neuropsychiatric disturbances accompanied by generalized chorea and was finally diagnosed with post-PV MF after a bone marrow examination. We also reviewed four cases of late PV associated with neuropsychiatric symptoms since 1966 and analyzed their clinical characteristics and therapeutic effects.

**Conclusion:**

Our case indicates that Janus kinase 2 (JAK2)-related PV is a treatable cause of late-onset chorea and that chorea may herald the deterioration of hematological parameters. Our case provides a clinically specific representation of post-PV MF. Patients with a long course of PV are recommended to undergo bone marrow re-examinations when they present with neuropsychiatric symptoms to achieve an early diagnosis of post-PV MF.

## Background

Polycythemia vera (PV) is a characteristic Philadelphia chromosome-negative myeloproliferative neoplasm (MPN) (1). Neurological manifestations of PV, such as headache, vertigo, visual disturbance, paresthesia, tinnitus, and transient ischemic attack, are frequent (50%–78%) (2). However, chorea and neuropsychiatric disturbances are rarely reported.

Post-PV myelofibrosis (MF) is a unique advanced stage in the natural progression of PV (3). The reported 10-, 15-, and 20-year incidences of post-PV MF transformation among patients are 27.4%, 39.9%, and 61.1%, respectively (4). Once PV develops into post-PV MF, the incidence of acute myeloid leukemia transformation is 1.6/100 persons/year, and mortality significantly increases to 7.4/100 persons/year (5). The overall survival of patients with post-PV MF has been reported to be 5.7–8 years (4). Therefore, early diagnosis of post-PV MF is important. However, apart from the routine re-examination of blood, bone marrow, and gene monitoring, there is no specific clinical characterization of the transformation to fibrosis in PV patients with a long disease course. The role of psychiatric disorders and chorea in post-PV MF has not yet been reported.

Here, we present the case of an elderly patient with JAK2^V617F^-positive PV who developed acute-onset reversible psychiatric behavior accompanied by generalized chorea and was finally diagnosed with post-PV MF after a bone marrow examination. We also review the literature regarding this disorder and discuss the possible mechanisms for the central nervous system (CNS) symptom burden of post-PV MF.

## Case presentation

A 68-year-old man was presented to the emergency department in November 2022 with an acute onset of psychotic derangement. He exhibited delirium, agitation, and slight involuntary movements involving the face, trunk, and left-sided limbs. These symptoms had suddenly developed a day earlier. The patient demonstrated, during admission, progressive behavioral changes characterized by sedentary inability, groping behavior, hallucinations, increased motor restlessness, night- and day-reversed sleep, and hypologia (Supplementary Video 1). The patient was diagnosed with JAK2^V617F^-positive PV confirmed by bone marrow biopsy 10 years previously and had been successfully treated with hydroxyurea 500 mg once daily since then.

In the past 3 years, the patient had two episodes of choreiform movements after hydroxyurea withdrawal. The movements were quickly relieved after symptomatic treatment with tiapride hydrochloride tablets. His hematological test results deteriorated, accompanied by spontaneous hematoma of the hip and infarct of the spleen, thought to be complications of PV. However, his bone marrow aspirations showed no progress or change. The hematological indicators during the past two episodes of choreiform symptoms are shown in Table 1. However, the patient continued to take hydroxyurea.

**Table 1:** Laboratory findings in the patient.

Investigation	2020–8	2022–02	2022–11	Reference values
White cell count	11.3 ↑	13.3 ↑	36.5 ↑	3.0–10.0 × 10^9^/L
Neutrophil ratio	0.670	0.731	0.700	0.40–0.75
Red cell count	6.82 ↑	6.91 ↑	4.98	4.3–5.8 × 10^12^/L
Platelet count	97 ↓	77 ↓	125	150–400 × 10^9^/L
Hematocrit	54.1 ↑	59.9 ↑	41.9	40–50%
Hemoglobin	166 ↑	181 ↑	114	115–155 g/L
Erythrocyte width	23.4 ↑	20.3 ↑	22.8 ↑	11–14.5%
Creatinine	103.3	121.4	275	49–92 μmol/L
C-reactive protein	63.43	5.32	41.52	≤6 mg/L
Presentation at onset	Involuntary movements involving four limbs, lips, and face	Involuntary movements involving the upper and right lower limbs, lips, and face	Involuntary movements involving the left-sided limbs and face; Neuropsychiatric disturbance	–
Concomitant symptoms/diseases	Spontaneous hematoma of the hip	Spleen infarction	renal insufficiency	–
Cerebral MRI	Multiple ischemic lesions in the right frontoparietal lobe and lateral ventricle; leukoaraiosis	Left frontal lobe and right parietal lobe softening foci; leukoaraiosis	Left frontal lobe and right parietal lobe softening foci with gliosis and signs of cerebral small vessel disease	–
Electroencephalogram	Negative	Negative	Mild abnormality	–
Use of hydroxyurea at the onset	Withdrawn for about four months	Withdrawn for about eight months	0.25 g once daily	–
Bone marrow examination	PV	PV	PV with fibrosis	–
JAK2 ^V617F^ mutation	+	+	+	–
Used therapies	Hydroxycarbamide 0.5 g qd; Tiapride hydrochloride 1#-1/2#-1#	Hydroxycarbamide 0.5 g bid; Tiapride hydrochloride	Clonazepam 1 mg qn; Hydroxycarbamide 0.25 g qd; Quetiapine 1/2 qn	–
Time to symptom relief	1 week	2 weeks	2 weeks	–

MRI magnetic resonance imaging; JAK Janus kinase; PV polycythemia vera.

The patient was not treated with chorea-inducing drugs such as antiparkinsonian drugs, tricyclic antidepressants, or anticonvulsants. There was no history of cognitive or behavioral issues. The patient’s family history was unremarkable. There was no medical history of peripheral vascular disease, metabolic or endocrine disorders, or autoimmune diseases.

General examination revealed facial erythrosis but no splenomegaly or hepatomegaly. The patient’s blood pressure was 130/80 mmHg, and his temperature was 36.6°C. Neurological examination revealed mild choreiform movements of the left limbs and orofaciolingual muscles with writhing tongue movements, pouting, and grimacing. Eye movements, muscle strength, and reflexes were normal. Neuropsychological testing was impossible to complete (secondary education). Other neurological tests showed no abnormalities or Parkinsonian, pyramidal, or cerebellar signs.

**Figure 1:**
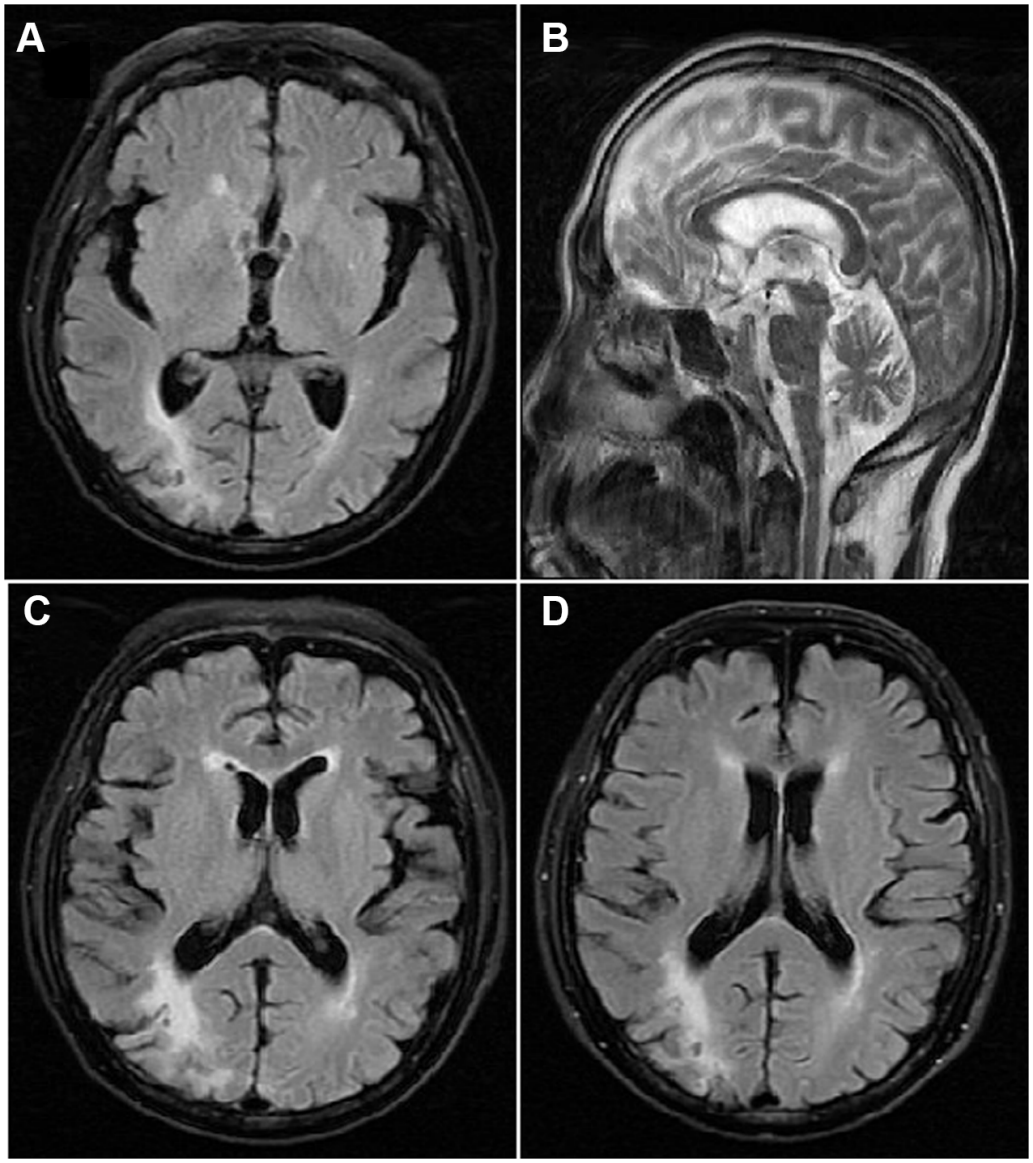
T2 weighted Axial view Magnetic resonance imaging showing **(A)** no infarct or space occupying lesion in the caudate nucleus, putamen or the globus pallidus, **(B)** and the third and fourth ventricle, and **(C)** encephalomalacia of the left frontal and right parietal lobes, gliosis, and signs of cerebral small-vessel disease; **(D)** encephalomalacia of the right parietal lobes at the second episode of chorea.

An extensive diagnostic laboratory workup for chorea and neuropsychiatric derangement was performed. The patient had a total white blood cell count of 36.5 × 10^9^/L, erythrocyte width of 22.8%, total red blood cell count of 4.98 × 10^12^/L, platelet count of 125 × 10^9^/L, hemoglobin of 11.4 g/dL, and hematocrit of 41.9%. The peripheral blood smear revealed no abnormal cells or acanthocytes. Results of serum liver enzyme, TSH, antistreptolysin O titer, ceruloplasmin, vasculitis workup, human immunodeficiency virus, syphilis serology, and autoimmune encephalitis panel were negative. His initial creatinine was 275 μmol/L, but it recovered quickly after fluid rehydration, which rarely caused renal encephalopathy. Chest and abdomen CT showed no obvious signs of systemic inflammation, tumor, and splenomegaly. The cerebral magnetic resonance imaging showed encephalomalacia of the left frontal and right parietal lobes, gliosis, and signs of cerebral small-vessel disease (Fazekas 2) (Figure 1). There were no acute infarcts, bleeding, or space-occupying lesions in the brain parenchyma, including the striatum, basal ganglia, subthalamic region, and brain stem. Electroencephalography revealed a mild abnormality, characterized by a background of low-medium amplitude 8-10c/s alpha wave rhythm, with slightly more low-medium amplitude 5-7c/s theta wave rhythm, without epileptic waves. Bone marrow examination revealed global hyperplasia with fibrosis, consistent with post-PV MF. JAK2^V617F^ mutation was detected in the peripheral blood, whereas the *BCR-ABL* gene was negative. All the above results demonstrated a diagnosis of chorea and neuropsychiatric derangement because of post-PV MF.

The dose of hydroxyurea was not increased, and it was not adjusted to a JAK2 inhibitor because of side effects and the limitation of thrombocytopenia. The severity of involuntary movement and mental and behavioral abnormalities gradually decreased, the involuntary facial and limb symptoms disappeared, and the patient could walk normally after 2 weeks of treatment with tiapride hydrochloride, clonazepam, and quetiapine tablets. A follow-up outpatient visit 6 weeks after his initial presentation showed a leukocyte count of 4.1 × 10^9^ /L, a hemoglobin level of 12.5 g/dL, a platelet count of 115 × 10^9^ /L, and an erythrocyte width of 11.9%. The chorea had completely subsided, and the tiapride hydrochloride tablet was slowly withdrawn. All sedating medications were discontinued, with no re-emergence of mental symptoms. The patient was referred to the hematology department for follow-up.

## Discussion and conclusion

Chorea, a rare but acknowledged PV symptom (6), has been described as an exacerbation of myeloproliferative disease (7), as in our case. In the two initial chorea episodes, laboratory tests revealed increased hematocrit, hemoglobin, erythrocyte count, mild leukocytosis, and decreased platelet count. The patient also developed complications caused by abnormal coagulation, including hip hematoma and splenic infarction, but bone marrow examinations were negative. Improvement in the clinical picture occurred simultaneously with normalizing hemoglobin and hematocrit levels. Our case confirms JAK2-related PV as a treatable cause of late-onset chorea, reveals that chorea may herald the deterioration of hematological parameters (7, 8), and highlights the importance of the standardized use of hydroxyurea.

The exact pathophysiology of polycythemic chorea remains unclear, and corticobasal ganglia circuitry perturbation (9) may be its anatomic basis. It is hypothesized to be based on hyperviscosity because of the raised red cell mass in the neostriatal area, probably led to a sluggish cerebral blood flow, venous stasis, impaired oxygen, and glucose metabolism (10), causing movement disorder. However, this has not been uniformly confirmed with functional neuroimaging studies (9–11). In our patient, the indicators of hematologic deterioration at his third chorea episode were significantly different from those of the previous two, mainly represented by peripheral leukocytosis. However, hematocrit and red blood cell count were normal, showing that the pathophysiological mechanism leading to this chorea symptom was not hyperviscosity alone.

Neuropsychiatric findings such as delirium, dementia, depression, mania, abulia, and frontal lobe syndrome in PV have been described in approximately 10 cases since 1960 (12–16). The currently reported numbers are probably underestimated because of the unfamiliarity of the link between specific neuropsychiatric symptoms and PV. The benefits of treatment appear ambiguous. In some cases, the mental disorder completely normalizes after phlebotomy and normalization of the hemoglobin levels, which supports the possible causal relationship between PV and the occurrence of these mental disorders (17–19). However, Mazzoli et al. (20) considered the possibility of a casual association between polycythemia and psychotic depression.

A literature search showed that most of the reported neuropsychiatric symptoms occurred in patients with PV who had been diagnosed many years ago, which is consistent with the natural course of PV. As a relatively indolent myeloid neoplasm, PV can progress to secondary MF, termed post-PV MF, and to the blast phase, worsening survival (21, 22). The median survival of patients with MF depends on the risk category and can range from 2 to 9 years, with hematopoietic stem cell transplantation being the only curative option (5, 23). The MYSEC (Myelofibrosis Secondary to PV and ET-Prognostic Secondary to PV and ET) database (5) showed that the longer the period between PV diagnosis and secondary myelofibrosis, the worse the survival. This finding indicates careful monitoring of patients with PV to identify the evolution of secondary MF earlier, especially if disease-modifying treatments are envisaged. The MYSEC Prognostic Model (5) considers constitutional symptoms, anemia, circulating blasts, thrombocytopenia, advanced age, and the absence of calreticulin mutations as risk variables. These variables may indicate the progression of a more indolent disease to an aggressive disease. Risk factors for overall survival include leukocytosis, venous thrombosis, and an abnormal karyotype. However, the clinical features that predict MF lack specificity; constitutional symptoms, including weight loss, night sweats, and fever, are easily overlooked. The neurological manifestations of post-PV MF mostly include thromboses (24), and some are intracranial extramedullary hematopoiesis (25). The role of psychiatric disorders and chorea in post-PV MF has not yet been reported. In this case, MF was detected by bone marrow puncture, we detected that the psychiatric symptoms may be a CNS manifestation of post-PV MF after excluding other common causes.

We reviewed four cases of late PV associated with neuropsychiatric symptoms since 1966, according to the clues above, and analyzed their clinical characteristics. The four reports (Table 2) describe four PV patients (two males and two females) with mental symptoms (13, 18–20). The mean age of the patients was 75.3 years (range 64–96), and the mean latency from PV to the development of mental symptoms was 10 years (range 9–11). Depressive symptoms were prominent, mental symptoms were severe, and treatments were difficult; one patient succumbed to absolute therapy-resistant depression (19). Chorea symptoms were present in two patients (13, 19). However, in one of them, the symptoms could not be distinguished from those related to the disease or psychotropic drug use (19). Three patients experienced various thrombotic events, including myocardial infarction, deep vein thrombosis of the lower extremities, TIA, and stroke. Laboratory examination indicated that the red blood cell count and hematocrit were nonspecific in patients at this stage, while two patients showed a leukoerythroblastic peripheral blood picture. According to the criteria (26) for post-PV MF, a diagnosis requires the presence of ≥grade 2 fibrosis accompanied by the development of progressive splenomegaly, anemia, leukoerythroblastosis, or constitutional symptoms. Splenomegaly and weight loss were present in three of the four patients, but no bone marrow examination or genetic testing was performed. In our case, bone marrow examination showed fibrosis (grade 2, on a 0–3 scale). Our patient also met another required criterion: medical history and two additional criteria: anemia and leukoerythroblastic peripheral blood image (26). He lacked splenomegaly and had none of the three constitutional symptoms. Neuropsychiatric symptoms and chorea were the most prominent clinical symptoms. Observing neuropsychiatric symptoms in PV should raise the suspicion of a fibrotic transformation.

**Table 2:** Summary of the findings in PV patients with mental symptoms after a long disease course.

Author, year	Sex, age	Latency from PV to mental symptoms (years)	Clinical features		Thrombosis	Routine blood tests		Bone marrow examination	Three constitutional symptoms: >10% weight loss in 6 months; night sweats; unexplained fever (>37.5°C)	Splenomegaly	Leukocytes	Anemia	Genetic testing	CT/ MRI	Treatment	Prognosis
			Mental	Chorea		RBC (10^12^/L)	Hematocrit (%)									
Murray et al. (18)	M, 72	11	Depression, Suicidal ideation, Auditory hallucinations, Paranoid delusions, Disorientation	–	Frequent TIA	*N*/A	Unknown	WNP	Weight loss (5 kg)	Yes	*N*/A	No	WNP	No focal lesion but generalized atrophy	Haloperidol and electroconvulsive therapy; lofepramine; fluoxetine	After 6 weeks, residual anxiety symptoms
Mazzoli et al. (20)	F, 69	9	Depression, Suicidal ideation Delusion	–	Two myocardial infarctions; frequent TIA; A stroke	9.9	Unknown	WNP	Weight loss	Yes	14	*N*/A	WNP	*N*/A	Busulfan and pentoxifylline	No effect on depression
Bauer (19)	F, 64	10	Severe depression; Temporary paranoid fears and delusions. Progressive cognitive impairment	Rhythmical trunk and pelvic movements; bucco-linguo-masticatory syndrome	Deep vein thrombosis of the leg	6.4	55	WNP	Considerable weight loss (20 kg)	Yes, Splenectomy	25.7	*N*/A	WNP	Normal	Busulfan; various antidepressant drugs; hydroxyurea; acetylsalicylic acid	Succumbed to absolute therapy-resistant depression
Garcia et al. (13)	M, 96	A long time	Progressive behavioral change, e.g., disinhibition, inappropriate verbal comments, unrestrained laughter	Sudden onset in left-sided chorea of the upper limb and face	None	*N*/A	46	WNP	*N*/A	No	*N*/A	No	WNP	Normal	Haloperidol and clonazepam Hydroxycarbamide	No effect; Subjective decrease in involuntary movements

CT, computed tomography; WNP, was not performed; MRI, magnetic resonance imaging; F, female; M, Male; N/A, unavailable data; RBC, red blood cell; TIA, ransient schemic ttack.

None of these patients with PV-related neuropsychiatric disorders underwent bone marrow re-examination before treatment. Managements of their psychiatric symptoms, including antipsychotic symptomatology and cytoreductive therapy by hydroxyurea, were ineffective, which may signal disease progression and provide an opportunity for a shift in treatment decision-making. Hydroxyurea is the most used first-line cytoreductive agent for the treatment of PV, which is not as effective as ruxolitinib in relieving neurological symptoms in PV (27). A phase 3 open-label study to evaluate the efficacy and safety of ruxolitinib versus standard therapy included hydroxyurea in patients with PV (27) found that the rate of improvement in the ruxolitinib group (49%) with a reduction of at least 50% in the 14-item Myeloproliferative Neoplasm Symptom Assessment Form total symptom score was significantly higher than in the standard therapy group (4.9%) at week 32. Unexpectedly, ruxolitinib-treated patients had greater reductions in almost all individual symptoms. In contrast, patients receiving standard therapy had more neurological symptoms, including ear ringing, concentration problems, numbness or tingling in the hands or feet, headache, and dizziness. A dysfunctional microcirculation caused by chronic inflammation might account for neurological symptoms, and relief of symptoms depends on the anti-inflammatory effect of ruxolitinib.

Chronic inflammation is a highly important driving force of MPN development and progression (28), in which the JAK–STAT-signaling and the NF-kB pathways are activated because of driver mutations and play a major role (26). Mutated blood cells are involved in the occurrence of microcirculation disorders through various pathways (28). In the PV stage, the increased number of RBCs contributes significantly to high blood viscosity in the cerebral microcirculation with increased vascular resistance and slowing blood flow (29). Red blood cells from patients with PV also have been shown to adhere more strongly to endothelial cells. Elevated hematocrit is associated with decreased cerebral blood flow and cerebral hypoxemia (30) and gives rise to abnormally high shear stress on the vessel wall, which may facilitate endothelial dysfunction. In MF, leukocytosis and thrombocytosis occur in the early hyperproliferative phase, and pancytopenia often occurs in the advanced stages (31). Elevated cell counts, activated circulating myeloid cells, and micro-aggregates of leukocytes and platelets (31, 32) may intermittently plug the cerebral microcirculation. However, a decrease in cerebral blood induces hypoxemia and activation of several signaling pathways, ultimately eliciting a neuroinflammatory state with microglia activation and induction of inflammatory cytokines (28). In our case, the corticostriatal basal ganglia circuit was disturbed by microcirculation disturbance caused by long-term chronic inflammation. The first two chorea symptoms occurred in the PV stage because of the dysfunction caused by blood viscosity caused by the increase of red blood cells, and hydroxyurea was effective (33). When the patient developed into the early stage of MF, leukocytosis and cytokine storm led to mental symptoms and simultaneously caused motor dysfunction by affecting cerebral blood flow in the basal ganglia.

Psychiatric symptoms in patients with JAK2-related PV over a long course of disease should be alerted to a disease progression. Most patients have non-low risks currently, according to multiple prognostic models to predict survival in MF (26). However, hydroxyurea is usually suitable for low-risk or intermediate-risk patients with platelet count <50×10^9^/L (26). We need to adjust the treatment strategy. The COMFORT trials demonstrate that ruxolitinib can improve splenomegaly and symptom burden and reduce cytoses and proinflammatory cytokine levels (34).

In conclusion, this is the first reported case of post-PV MF with chorea and neuropsychiatric symptoms. The onset of chorea has been linked to worsening hematological values and PV progression, and the resolution of chorea has been related to PV treatment. Importantly, this case provides a possible clinical representation of post-PV MF. It is recommended that patients with a long course of PV receive prompt bone marrow re-examination when they have mental symptoms to achieve an early diagnosis of MF and avoid delays that may shorten their survival.

## Data availability statement

The original contributions presented in the study are included in the article/Supplementary material, further inquiries can be directed to the corresponding author.

## Ethics statement

Ethical review and approval was not required for the study on human participants in accordance with the local legislation and institutional requirements. Written informed consent from the patients/participants or patients/participants' legal guardian/next of kin was not required to participate in this study in accordance with the national legislation and the institutional requirements. Written informed consent was obtained from the patient’s spouse for the publication of any potentially identifiable images or data included in this article. Written informed consent was obtained from the participant/patient(s) for the publication of this case report.

## Author contributions

LL: Conceptualization, Data curation, Writing – original draft. MZ: Formal analysis, Investigation, Writing – original draft. Y-QW: Data curation, Visualization, Writing – original draft. W-NF: Supervision, Writing – review & editing. DL: Writing – review & editing.
